# The Use of Artificial Neural Networks for Forecasting of Air Temperature inside a Heated Foil Tunnel

**DOI:** 10.3390/s20030652

**Published:** 2020-01-24

**Authors:** Sławomir Francik, Sławomir Kurpaska

**Affiliations:** 1 Department of Mechanical Engineering and Agrophysics, University of Agriculture in Krakow, 31-120 Kraków, Poland; 2 Department of Bioprocess, Power Engineering and Automation, University of Agriculture in Krakow, 31-120 Kraków, Poland; slawomir.kurpaska@urk.edu.pl

**Keywords:** artificial neural network, perceptron, temperature, forecasting, greenhouse, greenhouse foil tunnel

## Abstract

It is important to correctly predict the microclimate of a greenhouse for control and crop management purposes. Accurately forecasting temperatures in greenhouses has been a focus of research because internal temperature is one of the most important factors influencing crop growth. Artificial Neural Networks (ANNs) are a powerful tool for making forecasts. The purpose of our research was elaboration of a model that would allow to forecast changes in temperatures inside the heated foil tunnel using ANNs. Experimental research has been carried out in a heated foil tunnel situated on the property of the Agricultural University of Krakow. Obtained results have served as data for ANNs. Conducted research confirmed the usefulness of ANNs as tools for making internal temperature forecasts. From all tested networks, the best is the three-layer Perceptron type network with 10 neurons in the hidden layer. This network has 40 inputs and one output (the forecasted internal temperature). As the networks input previous historical internal temperature, external temperature, sun radiation intensity, wind speed and the hour of making a forecast were used. These ANNs had the lowest Root Mean Square Error (RMSE) value for the testing data set (RMSE value = 3.7 °C).

## 1. Introduction

Agriculture plays a critical role in the global economy. Agriculture development requires the introduction of new farming technologies. Digital agriculture (a new scientific field) uses data intense approaches to increase agricultural productivity, economic efficiency, and optimal use of scarce resources while minimizing its environmental impact. One opportunity to increase agricultural productivity is to use a protected and controlled environment such as greenhouse systems. The controlled environment of a greenhouse prolongs the cultivation periods of seasonal crops, increases yield, reduces transportation distances, decreases water consumption, land usage and reduces the need for pesticides. Greenhouse systems are an essential element of agricultural production. They are an important way to meet the growing food demand. Crops can be produced year-round regardless of the climate. Greenhouse cultivation is the most intensive way of producing mainly vegetables and flowers. Over one hundred countries use the agricultural greenhouses for the commercial vegetable production [1,2,3,4,5,6,7]. Additionally, for opencast agriculture, the change in land usage usually causes soil erosion [8,9,10,11,12]. For this reason, the agricultural solution on adverse climatic conditions, e.g., droughts and temporary frost, is the greenhouse microclimate.

Today, there is a great interest in using automation and electronics for monitoring and controlling all the processes on farms. An example would be monitoring and forecasting climate conditions. Scientific studies have shown that, for opencast agriculture, Fourier analysis of hourly data demonstrates that the atmospheric pressure intervened in the occurrence of rainfall on semi-arid regions [13,14,15,16]. The production management in greenhouse systems demands decision-making over several timescales. Decisions regarding controlling the internal environment of the greenhouse are particularly difficult. Climatic conditions inside greenhouses are often very complex and depend considerably on the external climatic conditions. Therefore, frequent and fast decisions are required for the system to be adaptable to alternating weather conditions. Maintaining the environmental conditions in greenhouse systems, for the appropriate quality and productivity of plant growth, involves monitoring and regulating temperature, relative humidity, carbon dioxide levels, light availability and water usage. Continuous monitoring of these environmental variables gives information to the horticulturalists on how to maintain maximal crop productivity. Controlling each of the variables affecting the internal environment of a greenhouse is a dynamic procedure, which requires the use of computers (computer Decision Support Systems—DSSs). It is important to correctly predict microclimate of a greenhouse for control and crop management purposes [2,6,17,18,19,20].

Greenhouses are energy-intensive compared to other sectors of the agricultural industry, therefore energy-efficient operation of a greenhouse is an important aspect. Reducing their energy consumption is one of the means to reduce the cost production. For effective decision-making, greenhouse control systems require energy models to accurately predict the thermal process in greenhouses [2,6,21,22]. Modelling the microclimatic parameters of a greenhouse is critical to optimize internal climatic conditions of greenhouses during different stages of plant growth. The basic problem is to develop an accurate model [7,19,23,24].

The greenhouse environment is a very complex, nonlinear, multi-input and multi-output dynamic system. It is a complex thermodynamic system—the internal greenhouse climate is a function of heat and mass transfer. This system is described by many interconnected variables such as light, temperature, relative humidity and carbon dioxide concentration. There are also time delays and intensive disturbances from the surroundings. This makes establishing an accurate mathematical model of a greenhouse’s microclimate intricate and difficult [2,4,6,18,20,24].

A great deal of research has been done in greenhouse modeling in the recent years. Many models have been developed to predict energy consumption and greenhouse microclimatic parameters. The internal greenhouse conditions depend on three major variable types, namely weather variables, control variables, and state variables. Thus, it is necessary to develop a suitable greenhouse model to control microclimatic variables [25]. One such type of a model are simulation models that allow for correctly predicting the internal temperature as a function of external climate parameters and greenhouse characteristics [6,20,23,26].

Accurately predicting temperatures in greenhouses has been a focus of research as internal temperature is one of the most important influencing factors for crop growth and development. Therefore, it is important to set up a precise predictive model of temperature that can predict temperatures several hours in advance. The modeling method must be successful enough to accurately forecast the greenhouse temperature with incomplete information [4,18,27]. For example, time series analysis techniques and auto regression models have been implemented to predict the internal temperature of a greenhouse. The model parameters, external air temperature, external air relative humidity, external solar radiation and cloud cover used as inputs were found to be sufficient to predict the internal temperature of a greenhouse [18].

Weather forecasting is useful, as it has several applications and it may provide basis for many human endeavors. For example, agriculture is often extremely sensitive to climate change. Weather predictions are meaningful for producers to make decisions regarding activities and property protection. Areas such as high-precision vehicle localization, energy, transportation, aviation, and inventory planning can also clearly take advantage of weather predictions [28]. External climate parameters and their forecast are also used in many varying branches of science (e.g., wind farm energy production, epidemiology and cooling systems).

Wang et al. [29] conducted research with the purpose of developing a mathematical model for wind speed forecasts. Accurate wind speed forecasts play an important role in reducing uncertainty of wind power and enhancing utilization efficiency of wind energy. It is also essential to enhance the ability of wind speed forecasting for wind farm management systems. The simulation results demonstrate that the developed, improved, innovative hybrid model for wind velocity forecasting (which consists of a feature selection module based on empirical wavelet transformation–Kullback–Leibler divergence, a long memory extraction module based on autoregressive, fractionally integrated moving average, an input–output determination module based on phase space reconstruction, an optimization module based on modified bat algorithm with cognition strategy and a forecast module based on improved Back-propagation (BP) Neural Network) outperforms several common benchmark models (Back-propagation Neural Network, Extreme Learning Machine, Kernel Ridge Regression, least squares support vector machines, Naïve technique, improved Back-propagation Neural Network, and Empirical Wavelet Transform).

Climate and weather influence population health through a number of interrelated pathways. The growth, survival and dispersal of microorganisms is determined by environmental and hydrometeorological conditions. Research is being carried out to elucidate the interactions and biological mechanisms through which weather influences health. For example, Colston et al. [30] conducted research with the aim of selecting climate data and assessing their performance as predictors of rotavirus infections.

Li et al. [31] devised the Following Day Load Simulation (FDLS) method using weather forecast parameters, and a simulated test was carried out on an office building with an ice storage cooling system. Accurate simulation and forecast of cooling load is vital in the design of efficient cooling in a cold storage system. The accuracy of FDLS was validated by comparing the simulation results with the actual data collected from the cold storage building.

Xing et al. [32] proposed a new data-driven model based on the support vector machine. Data-driven models were developed and achieved good accuracy in monthly and daily soil temperature predictions of a single site taking air temperature, solar radiation and time as inputs. The new model considers daily soil temperature variations as superposition of annual average ground temperature predictions (long-term climate impact) and daily ground temperature amplitude predictions (short-term climate impact).

In recent years, different computational approaches were applied for temperature prediction. The temperature prediction methods can be divided into physical methods based on mathematical theory and black box methods based on modern computational technology [27]. The former group of methods is also based on modern computational technologies. Generally, the physical methods have a high degree of complexity with many parameters that have to be determined. It is often a difficult task to measure the parameters or get them from the sub-models. In contrast to physical methods, the ‘black box’ prediction methods don’t need to determine every parameter value [33]. As the latter group of methods are extremely effective, they are used to find answers to many research problems, though without being able to explain the system structure.

For finding dependencies among individual values describing air temperature inside a heated object, it is possible to use methods of Artificial Intelligence. Artificial Neural Network (ANN) is one such method. 

The term Artificial Neural Network refers to computational and machine learning techniques inspired by the functions of biological neural networks. ANNs were designed to simulate the behavior of the human neural system based on the relationships between input information and the target. A biological neural network consists of many interconnected biological neurons. ANNs are formed by simple units of processing, called neurons [34,35,36,37]. 

One type of neural networks is Multilayer Perceptron Neural Networks (MLPs). An MLP consists of the input layer, the hidden layer (one or two) and the output layer. Each layer consists of neurons with a nonlinear activation function, which are connected to each other. MLP learning is based on a gradient descent method that minimizes the sum of squared errors between actual and desired output values [38,39].

ANNs are powerful computational models. ANNs are also universal nonlinear approximators and can detect relationships between input and output variables, even if they are complex or unknown. ANNs are able to describe any nonlinear function given enough hidden units and learning data. ANN is a robust data modeling tool that is widely used in many applications, such as regression, classification, and approximation-based learning processes. Implementation of Artificial Neural Networks for different research proved to be very efficient and accurate. ANNs are characterized by very high accuracy in comparison with other mathematical models (regression models, general linear models, regression trees) [1,5,6,35,36,40,41,42,43]. For example, Kasantikul et al. [36] proposed a combination of an ANN and a particle filter (PF) to estimate wind speed. The overall root mean-square error of different ANN techniques (the ANN based PSO, meaning Particle Swarm Optimization combined with PF, the ANN based BP, and the ANN based PSO) is better than the regression.

Nonlinear models, especially ANNs, are also increasingly used in agriculture, often obtaining better analysis results than classical statistical methods [44].

Castaneda-Miranda and Castano [45] used an MLP for frost control in a greenhouse. The error values of the best models: ARX (the autoregressive models with external input) and ANN for the summer and the winter seasons were compared. ANN models obtained less Standard Error of Prediction (SEP) and Mean Absolute Percentage Error (MAPE) than ARX models.

ANNs are widely employed to exploit the empirical knowledge. Particularly, ANNs are applied in modelling, classification and clustering tasks, prediction processes, decision-making processes and management of industrial production systems [46]. ANNs are used in various scientific fields including, for example, bioinformatics, biochemistry, medicine, meteorology, economic sciences, robotics, aquaculture, food security and climatology. ANNs are also used in agriculture, agrophysics or agricultural engineering [1,35,40,41,42,43,47].

All applications of ANNs can be classified into six typical groups of techniques: association (for reducing data dimensionality), classification (for grouping data into classes), conceptualization (for conceptualizing ideas based on concrete data), prediction (for predicting values), optimization (for seeking convergence to a minimum or maximum), and filtering (for sifting data according to restrictions) [48].

One of the tasks that can be performed by an ANN is prediction. This task is used to assist in the control and monitoring of numerous variables in several areas [37]. Many ANN applications are related to renewable energy sources (different uses of ANN models for better energy production predictions). Research addresses, for example, the use of ANNs to forecast solar radiation (the main problem for the best use of photovoltaic systems) and wind power forecasting [37,38,48,49,50]. ANNs are applied for forecasting building energy usage and demand [34]. The ANN for the prediction of odor properties of post-fermentation sludge from a biological-mechanical wastewater treatment plant was developed. The ANN comprised of four layers: input layer (eight neurons), two hidden layers (four and two neurons) and output layer (one neuron) [51]. In medicine, ANNs are used to predict mortality risk and incidence of disease. In the area of railway engineering, studies have predicted failure points in rail turnouts and the rates of wear of wheels and rails [37]. Several works investigate an ANN approach to paper-making (mainly addressing the application of neural models for paper properties’ prediction). An ANN was implemented to predict the fibres’ length as a function of the refining processes’ variables. The prediction of this parameter is crucial to obtain a high-performance process [46]. Another example is a neural network model that was created for predicting tool wear and surface roughness during turning. The validation using neural networks gave better results than the one using regression models. The developed forecasting system was able to accurately predict surface wear and roughness [52]. In the fault prognosis context, ANNs are important tools as they enable the implementation of the prediction task easily and accurately [37].

Many applications of ANNs involve forecasting and prediction in agriculture. The MLP neural network, with one hidden layer, was developed to predict the normalized undrained shear strength of organic soils. The MLP neural network, with an architecture of 5–4–1, provided a more reliable prediction in comparison to the empirical methods [53]. The ANNs with MLP topology to forecast winter rapeseed yields and winter wheat yield were developed. Using ANNs makes it possible to obtain an accurate yield forecast before harvesting (all tested models were characterized by high forecast accuracy) [39,44]. ANNs are also employed to control and predict variables in agricultural drying processes. For example, scientists, using an ANN, designed a system to identify the dynamics of the drying process in a rotary dryer, developed a tomato drying model, developed a system for predicting variables related to tobacco and willow woodchips’ drying processes [42,54]. The diversification of ANN applications is large, demonstrating the importance of this tool. They achieve high accuracy, are computationally efficient, and require no knowledge of the physical relationships between inputs and outputs. ANNs are a powerful tool for making predictions based on a large number of interrelated experimental data [5,6,21,27,40,41,42,43,54,55,56].

As the greenhouse system is nonlinear and variable in time, ANNs have been applied to greenhouse environment modeling, including internal temperature. For the first time, ANNs were used to predict greenhouse temperatures in the early 1990s [24,57,58]. Ferriera at al. [59] have used ANNs for determining the temperatures inside the greenhouse. They have taken into consideration solar radiation, both internal and external temperature and internal relative humidity. Linker and Seginer [60] used three models of ANNs for modeling the temperature inside of a greenhouse, namely: a model with a sigmoid function, a physical model with a hybrid function and a sigmoid model with utilization measured in situ value of meteorological data. Uchida Frausto and Pieters [61] have used an ANN for modeling temperatures inside of a greenhouse as a function of external temperature; air humidity; radiation intensity, and cloud state. They have ascertained on the base of analysis that the number of neurons, hidden layers and iterations renders important influence on accuracy of approximation. The variation of internal air temperature in a tropical greenhouse was investigated. The study included an auto regressive model with an external input, an auto regressive moving average model with an external input and a neural network auto regressive model with an external input. The model parameters—external air temperature, external air relative humidity, external solar radiation and cloud cover—used as inputs were found to be sufficient to predict the internal temperature of the greenhouse [18]. Dariouchy et al. [62] developed an ANN and used it to predict the internal temperature and the internal moisture inside the greenhouse starting from the external climatic data. This model can perform better than the time series prediction models. Trejo-Perea et al. [63] employed a MLP model validated with measured data and a nonlinear regression model for the prediction of greenhouse electricity usage. He and Ma [33] developed an ANN model for modelling air humidity inside a greenhouse in northern China during the winter period. A Back-propagation Neural Network based on principal component analysis was proposed for modeling the internal humidity of a greenhouse. An ANN was developed in order to predict the temperature and relative humidity in a greenhouse [64]. The previous values of the external temperature, the external relative humidity, command of heater and ventilator, and the previous values of internal temperature and internal relative humidity were defined as inputs. The current internal temperature and the internal relative humidity were considered as the outputs. This work demonstrates the applicability of the neural networks to model the internal air temperature and relative humidity in a greenhouse. Castaneda-Miranda and Castano [45] used a MLP for frost control in a greenhouse. Its ability to predict temperatures within control offers the benefit of being easy to implement without the need for a complex mathematical model a priori. 

As it can be seen from quoted research results, researchers used neural networks for engineer forecasting in agricultural objects. The purpose of our research was elaboration of a model that would allow to forecast changes of temperatures inside the heated foil tunnel using ANNs.

## 2. Materials and Methods

The literature survey and our experience obtained during the creation of neural models allowed to identify several characteristic stages occurring during this process [20,25,37,39,40,41,42,44,45,52]. The creation of an ANN model proceeds in five stages:

1. Formulating a semantic model.
2. Gathering experimental data.
3. Selecting neural network’s type and architecture.
4. Carrying out the process of networks’ learning.
5. Choosing and assessing the best model.

### 2.1. Formulating a Semantic Model

The creation of a forecast model begins with adopting a semantic model—conceptual model. Such model should logically resemble the theory describing modelled phenomenon in the form of a system of objects and relations between them. In case of the absence of theoretical assumptions, the experiment is used. In the case of forecast models, the development of the semantic model requires the selection of forecast variables, characterizing the forecasted phenomenon, and interpretative variables. Frequently, the determination of time horizon’s length, meaning the period of time for which the forecast is made, and the determination of historical data analysis period’s length is required.

In the case of the heated foil tunnel, internal temperature is the effect of heat transfer with the environment. From the heat transfer theory derives the fact that the covers’ insulation, surrounding climate’s parameters and required temperature inside the facility are the factors deciding the intensity of heat transfer between the interior of the facility and its environment. The factor summarizing the intensity of transfer is the rate of heat permeation through the cover, which is dependent on e.g., air velocity around the cover [65]. On the other hand, the heat transfer and the intensity of solar rays’ permeation through the transparent cover of the facility is dependent not only on the type of the cover but also on the shape of the roof [66].

As a result, in the paper, for the analysed type of the facility, for the forecast of internal temperature (a dependent variable) the intensity of solar radiation (S), temperaturę of the environment (Tex), wind velocity (W) and the heater temperature (Th) were taken into account as independent variables. Similar variables were adopted by other authors for forecasting the greenhouse microclimate’s parameters [21,23,27,45,62].

The values of external climate’s parameters (Tex, S, W) change over time randomly. In case of solar radiation and external temperature intensity, apart from random deviations, periodical change during the day and throughout the whole year can be detected. Therefore, for each adopted independent variable, several historical values (Tex, Th, S, W and Tin) were taken into account in the model. As the independent variables (inputs of the ANN), the date and hour of forecast was also adopted. It was assumed that this model would enable the forecast of temperature 1 to 4 h in advance (time horizon of forecast *t*_HP_= 1, 2, 3, 4).

As the base for creation of the prognostic model, it accepts the hypothesis:

(1) $$Tin\left(t_{0}+t_{HP}\right)=f\;\left\{\;D\;\left(t_{0}\right)\;,\;t_{0}\;,\;t_{HP}\;,\;Tex\;\left(t_{0-i}\right)\;,T\;\left(t_{0-i}\right)\;,\;S\;\left(t_{0-i}\right)\;,\;\;W\;\left(t_{0-i}\right)\;,Tin\;\left(t_{0-i}\right)\;\right\},$$

where:

*Tin*(*t*_0_ + *t*_HP_)—forecasted internal temperature for moment *t*_0+HP_, [°C]
*t*_0_—moment (hour), in which prediction was executed, [h]
*t*_HP_—time horizon of prediction; *t*_HP_ = 1, 2, 3, 4, [h]
*D*(*t*_0_)—next day of year, in which prediction was executed
*Tex*(*t*_0−*i*_)—external temperature in moment (*t*_0−*i*_), [°C]
*Th*(*t*_0−*i*_)—temperature of heater in moment (*t*_0−*i*_), [°C]
*S*(*t*_0−*i*_)—sunny radiation intensity in moment (*t*_0−*i*_), [W⋅m^−2^]
*W*(*t*_0−*i*_)—wind speed in moment (*t*_0-*i*_), [m⋅s^−1^]
*Tin*(t_0−*i*_)—internal temperature in moment (*t*_0−*i*_), [°C]
*i*—slip index in time; *i* = 0, 1, 2, 3, 4, 20, 21, 22, 23, [h].

### 2.2. Gathering Experimental Data

Experimental research was carried out in a heated foil tunnel situated on the property of the Agricultural University of Krakow. The experimental foil tunnel with dimension length × width: 9 m × 6 m was equipped with a standard heating system. Foremost walls of tunnels were covered with polycarbonate, with thickness of about 6 mm, and the arc part was covered with polyethylene foil, with thickness of about 0.18 mm. The total protection surface was 113 m^2^. Polycarbonate plates were fastened with T profiles. Heating installation consisted of two rows of heaters (with diameter of 75 mm) with total heating surface of circa 19 m^2^. Heating of hot water proceeded through an installed electric heater in the flow-heater.

During the experiments, sensors for measuring the heating water temperature and internal air temperature were installed in the researched tunnel. Outside of the tunnel temperature, air humidity, sun radiation intensity and wind speed were continuously measured. Monitored parameters were sent by a check-measuring system and recorded in a PC computer. During the research in the tunnel, work conditions (work–break cycle) were registered for the heater. Obtained results served as data for ANNs.

**Figure 1:**
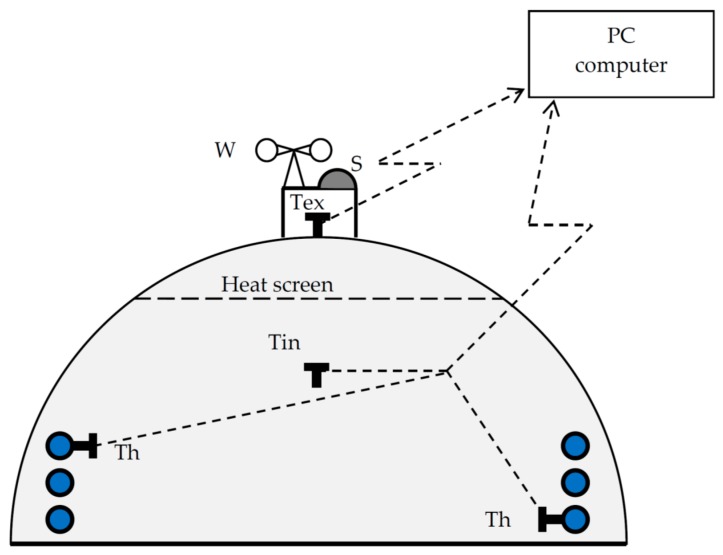
Scheme of the research stand.

The scheme of the research object is presented in Figure 1.

### 2.3. Selecting Neural Network’s Type and Architecture

The learning process of a neural network requires extracting subsets containing learning patterns and patterns used to assess the quality of the neural network (test and, possibly, validation patterns) from the data set (time series).

Data obtained from measurements were prepared for the neural network creation. Results of independent variable measurements executed from the 2nd to the 29th of April and from the 1st of October to the 2nd of November (1364 pattern) were randomly divided into three sets: learning (744 pattern), validation (312 pattern), and testing (312 pattern). 

Two different types of Feed-Forward ANNs (Radial Basis Function networks and MLP networks) were researched as the best suitable for forecasting temperature.

Establishing the ANN architecture requires determining the number of layers and the number of neurons in hidden layers. A larger number of layers and neurons in those allows for building a more complicated model but requires more learning patterns.

### 2.4. Carrying out the Process of Networks’ Learning

For creating the models and the learning process of ANNs, the function “Automatic Designer” of Statistica was used. 

The Back-propagation Learning Algorithm and then the Conjugate Gradient Algorithm (CGA were used for each ANN. Both of these algorithms are supervised learning methods. A supervised learning method requires a set of training patterns and their corresponding desired outputs. During the learning process, the algorithm corrects the connection weights among neurons according to imposed learning rules. As a result, ANNs obtain knowledge from the learning data. The Back-propagation Algorithm (BPA) is the best known learning method for neural networks and one of the most useful. It has lower memory requirements than most algorithms and usually reaches an acceptable error level quite quickly, although it can then be very slow to converge properly on an error minimum. The CGA is an advanced method of learning. It usually performs significantly better than BPA and can be used wherever the latter can be. BPA adjusts the network weights after each case, whereas CGA works out the average gradient of the error surface across all cases before updating the weights once at the end of the epoch.

During the learning process, 100 networks of different architectures were analysed, from which the best ten models were maintained.

### 2.5. Choosing and Assessing the Best Model

The value of root mean-square error (RMSE) calculated for testing data set was the criterion of choice. RSME is a commonly used statistical error to evaluate the model’s performance [36,39].

The RMSE was calculated from formula:

(2) $$RMSE=\sqrt{\frac{1}{n}\sum_{i=1}^{n}{\left(T_{me,i}-T_{cal,i}\right)}^{2}}$$

where:

*T_me,i_*—measured value of internal temperature, [°C]
*T_cal,i_*—calculated by ANN value of internal temperature, [°C]
*n*—number of observations.

## 3. Results

Preliminary research showed that the best forecasts were obtained with three- and four-layers’ MLP type networks. As a result of using Automatic Designer 10, networks of three-layer MLPs were developed. Characteristics of the networks are presented in Table 1.

**Figure 2:**
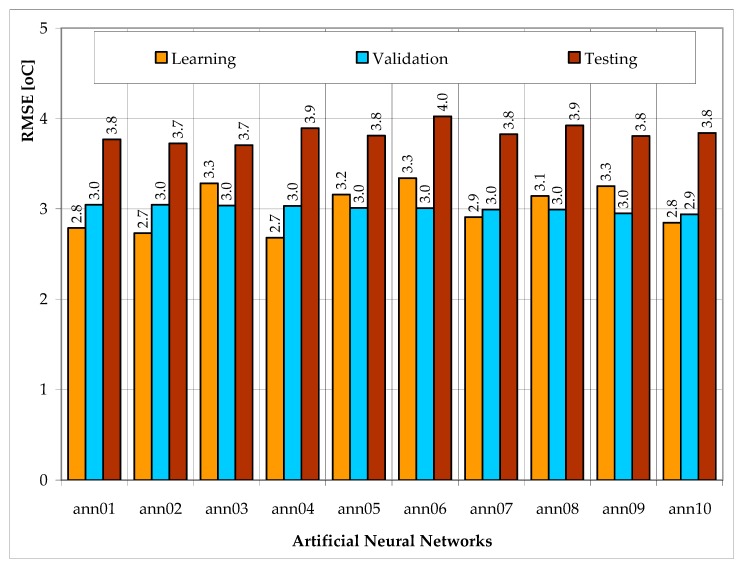
Values of RMSE for learning, validation and testing data sets.

In Figure 2, RMSE values for learning, validation and testing data sets are presented.

Taking into consideration all forecasts ascertained that networks ann02 and ann03 most accurately predict temperature inside of the tunnel. These two ANNs had the lowest error value for the testing data set (RMSE = 3.7 °C). RMSE values for other networks that ranged from 3.8 °C to 4.0 °C (for the testing data set). Neural models ann02 and ann03 had also the lowest error value for the validating data set (RMSE = 3.0 °C).

Finally, as a model for forecasting, the internal temperature ann02 network has been chosen on account of the smallest value of error for teaching data—for ann02, RMSE has reached a value of 2.7 °C and for ann03 RMSE = 3.3 °C.

Table 2 summarizes the RMSE values for different forecasting time horizons (*t*_HP_ = 1, 2, 3, 4 h).

**Figure 3:**
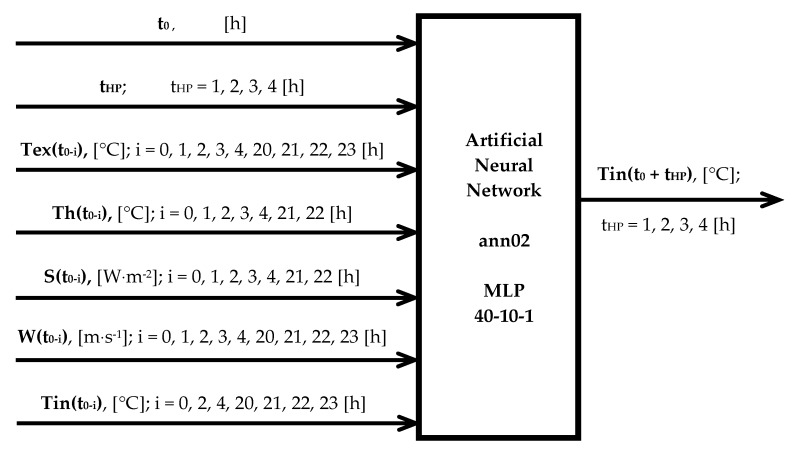
Structure of the ANN model.

In Figure 3, the structure of chosen neural model is presented. Neural network ann02 is a three-layer MLP with 10 neurons in the hidden layer and one neuron in the output layer. Values of forty variables served as the input. On the output of neuron network, the forecasted internal temperature for assigned forecast time horizon is obtained.

**Figure 4:**
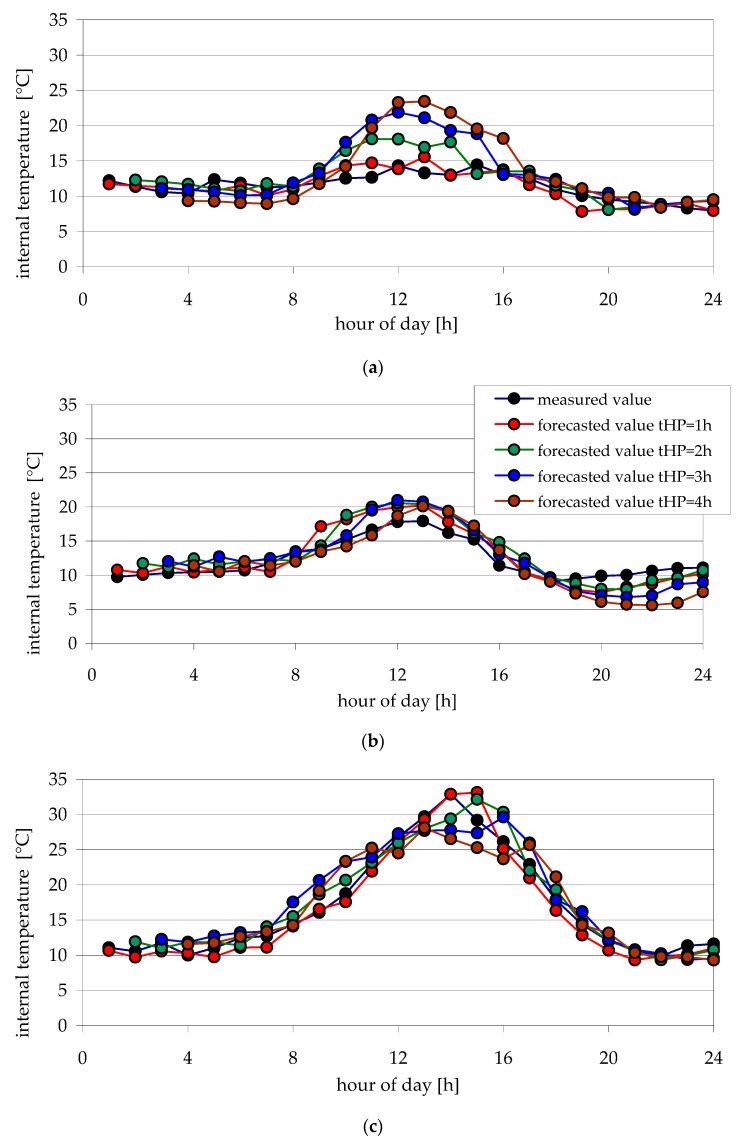
Forecasted and measured values of internal temperatures for selected days of April (test data) for various forecast time horizons: (**a**) 3th April; (**b**) 8th April; (**c**) 19th April.

**Figure 5:**
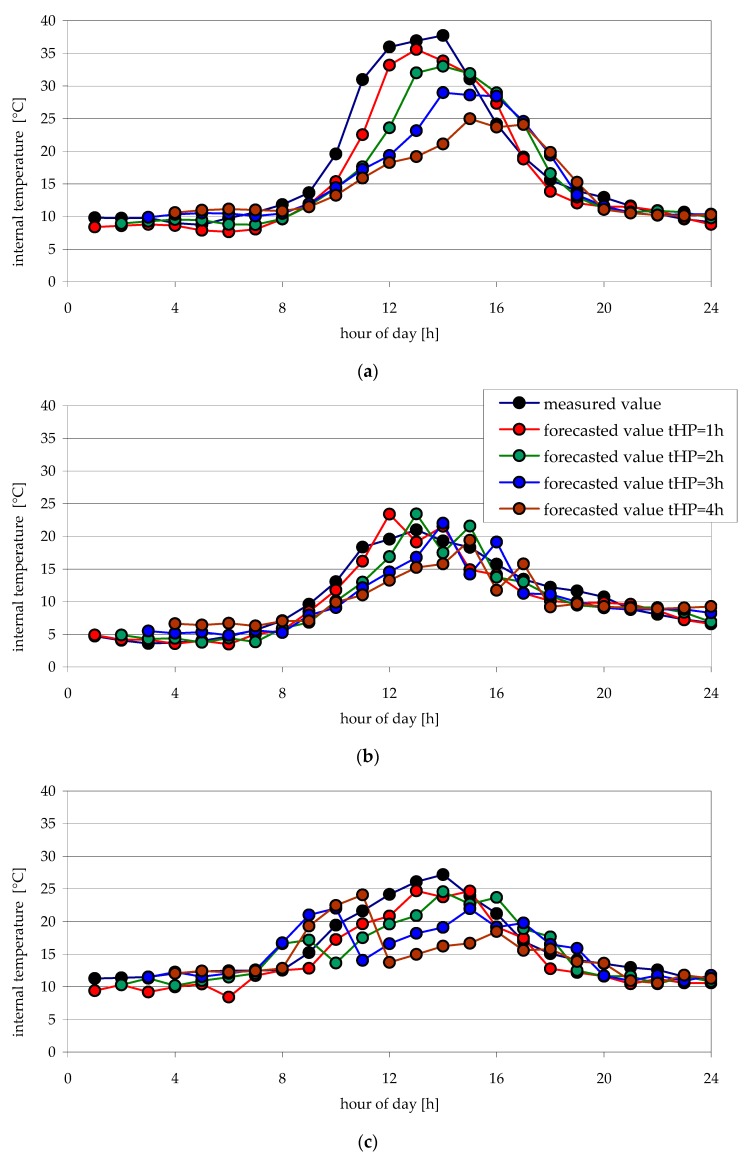
Forecasted and measured values of internal temperatures for selected days of October (test data) for various forecast time horizons: (**a**) 3rd October; (**b**) 14th October; (**c**) 29th October.

Figure 4 and Figure 5 show examples of internal temperature changes obtained from measurements and forecasts, for the time horizon of the forecast from one hour to four hours. Forecasts were made for testing data—examples of days: April 3rd, 8th and 19th, and October 3rd, 14th and 29th.

## 4. Discussion

Devised neural models maintained the ability to generalize obtained knowledge. RMSE values for all 10 neural models (Figure 2) evaluate to the highest values for the testing data set (RMSE = 3.7 ÷ 4.0 °C), which were not used to create neural models. Significantly lower values were obtained for data from the learning data set (RMSE = 2.7 ÷ 3.3 °C) and validating data set (RMSE = 2.9 ÷ 3.0 °C). Such result is in line with the theory of ANNs. The increase in error value for tested data is marginal, which is indicative of devised neural models maintaining the ability to generalize. If the difference was greater, it would mean that overlearning occurred. Overlearning is the result of an overly complex structure of a neural network compared to the amount of learning data. For a chosen neural model, the ann02 RMSE value for tested data was 36% higher in comparison with learning data and 22% higher compared to validating data.

After analyzing RMSE values for different forecast time horizons (Table 2), it can be stated that, with an increase in t_HP_, the RMSE value also increases. Error values for all devised ANN models for tested data amount to: RMSE = 2.5 ÷ 3.0 °C (for *t*_HP_ = 1 h), RMSE = 3.3 ÷ 3.6 °C (for *t*_HP_ = 2 h), RMSE = 3.9 ÷ 4.3 °C (for t_HP_ = 3 h), RMSE = 4.5 ÷ 5.0 °C (for t_HP_ = 4 h). Error for learning data is, respectively: RMSE = 2.0 ÷ 2.8 °C (for *t*_HP_ = 1 h), RMSE = 2.5 ÷ 3.0 °C (for *t*_HP_ = 2 h), RMSE = 2.7 ÷ 3.4 °C (for *t*_HP_ = 3 h), RMSE = 3.2 ÷ 4.2 °C (for *t*_HP_ = 4 h). In addition, for validating data: RMSE = 2.2 ÷ 2.5 °C (for *t*_HP_ = 1 h), RMSE = 2.7÷3.0 °C (for *t*_HP_ = 2 h), RMSE = 3.1 ÷ 3.3 °C (for *t*_HP_ = 3 h), RMSE = 3.4 ÷ 3.7 °C (for *t*_HP_ = 4 h).

For a chosen neural model, the ann02 RMSE value for testing data set increased with higher *t*_HP_ values, respectively: 2.5 °C → 3.4 °C → 4.1 °C → 4.6 °C. The increase in RMSE values for the learning data set, along with an increase in forecast horizons, progresses as follows: 2.0 °C → 2.5 °C → 2.8 °C → 3.4 °C. However, for the validating data set, RMSE values amount to 2.3 °C → 3.0 °C → 3.3 °C → 3.5 °C. 

Proven decrease in forecast accuracy with an increase in forecast time horizon is in line with the theory. The impossibility to predict interferences of forecasted system causes the inversion of trends present at the moment of making the forecast, which causes very rapid increase in forecast error. In the case of heated foil tunnels, such quickly changing system parameters are wind speed and sun radiation intensity.

The analysis of forecasted air temperature values inside the foil tunnel obtained using the chosen best neural model ann02 allows for concluding that they do not significantly stray from intended values (Figure 4 and Figure 5). For forecasts made for chosen days in April, only for the 3rd of April (Figure 4a) are greater differences for forecast time horizons of the 3rd and 4th hours. For October, however, the largest differences between the forecasted and measured internal air temperature are present on the 3rd of October for forecast time horizons of the 2nd, 3rd and 4th h (Figure 5a).

It is noteworthy that the greatest differences between forecasted and measured air temperature values are present from 10:00 a.m. to 4:00 p.m. (Figure 4a, Figure 5a–c). For night hours, however, forecast accuracy is very good. It is probably due to the influence of rapidly and chaotically changing sun radiation intensity on the temperature inside the tunnel.

After comparing internal temperature forecast errors for different forecast time horizons, it was stated that, for *t*_HP_ = 1 h and *t*_HP_ = 2 h devised ANN, ann02 accurately predicts the temperature. The differences between forecasted and measured temperature inside the tunnel do not exceed several degrees centigrade. Therefore, the devised neural model can be used as a supporting element for decision making in automatic temperature regulating systems.

ANNs forecasting temperature changes in the heated foil tunnel are used to devise a Decision Support System for heating control. The system would react in advance according to predicted temperature changes, which allows for saving energy used to heat the tunnel.

## 5. Conclusions

Conducted research confirmed the usefulness of ANNs as tools for making internal temperature forecasts. RMSE values didn’t exceed 4 °C (RMSE = 2.7 ÷ 3.3 °C for learning data, RMSE = 2.9 ÷ 3.0 °C and RMSE = 3.7 ÷ 4.0 °C for testing data). The increase in error value for tested data is marginal, which is indicative of the devised neural models’ maintained ability to generalize (the overlearning phenomenon does not occur). Therefore, it can be stated that the number of neurons in hidden layers was selected correctly and the learning data were sufficient. 

From all tested networks, the best is the three-layer MLP type network with 10 neurons in the hidden layer. This network has 40 inputs and one output (prediction of internal temperature). As the networks input previous historical internal temperature, external temperature, sun radiation intensity, wind speed and the hour of making a forecast were used. Other developed models were only slightly worse (had slightly higher RMSE value).

The ANN with forecast horizon of 1 h most accurately describes the course of temperature inside of the tunnel. An increase in prediction time increases the differences between measured and calculated values of temperature. In the case of prediction time horizon equal to 1 h, maximum absolute differences in forecasted temperature are within the range of 0 to ca. 7 °C. RMSE values for *t*_HP_ = 1 h amounted respectively to 2.5 °C (for testing data), 2.0 °C (for learning data) and 2.3 °C (for validating data). With an increase in *t*_HP_, the error in forecasted RMSE values also increased. For example, for testing data with t_HP_ increasing from 1 to 4 h, RMSE values changed as follows: 2.5 °C → 3.4 °C → 4.1 °C → 4.6 °C. We ascertained that the ANN model allows for generating accurately enough temperature forecasts up to two hours in advance.

The neural model forecasting temperature changes inside the heated foil tunnel obtained as a result of conducted research enables the optimization of decisions regarding the control of heating system. Consequently, this allows for lowering the energy usage needed to ensure an optimal internal climate in a heated foil tunnel. The ANN model can be also a part of a more complex DSS designated for internal climate management. Such systems are currently a subject of interest of many researchers and the number of scientific papers describing varying uses of even more accurate DSSs increases from year to year.

## Figures and Tables

**Table 1:** Characteristics of neural models.

ANN Model	Type of Network	Number of Inputs	Number of Neutrons in Hidden Layers
ann01	MLP 32-8-1	32	8
ann02	MLP 40-10-1	40	10
ann03	MLP 15-8-1	15	8
ann04	MLP 31-10-1	31	10
ann05	MLP 26-7-1	26	7
ann06	MLP 18-9-1	18	9
ann07	MLP 16-8-1	16	8
ann08	MLP 13-6-1	13	6
ann09	MLP 8-8-1	8	8
ann10	MLP 22-6-1	22	6

**Table 2:** RMSE values for different forecast time horizons.

Data Set	ANN Model	*t*_HP_ = 1h	*t*_HP_ = 2h	*t*_HP_ = 3h	*t*_HP_ = 4h
Learning	ann01	2.10	2.54	2.93	3.41
	ann02	2.01	2.54	2.81	3.38
	ann03	2.63	2.95	3.38	4.01
	ann04	2.15	2.48	2.74	3.23
	ann05	2.37	2.79	3.22	4.01
	ann06	2.75	3.01	3.36	4.10
	ann07	2.19	2.68	3.00	3.59
	ann08	2.34	2.91	3.34	3.80
	ann09	2.21	2.93	3.40	4.16
	ann10	2.16	2.67	3.02	3.39
Validation	ann01	2.36	2.87	3.17	3.63
	ann02	2.28	2.97	3.30	3.49
	ann03	2.47	2.83	3.13	3.60
	ann04	2.37	2.78	3.17	3.66
	ann05	2.40	2.78	3.10	3.63
	ann06	2.50	2.83	3.08	3.53
	ann07	2.49	2.90	3.12	3.39
	ann08	2.45	2.74	3.15	3.50
	ann09	2.19	2.81	3.13	3.51
	ann10	2.36	2.68	3.08	3.50
Testing	ann01	2.57	3.41	4.12	4.65
	ann02	2.53	3.39	4.07	4.59
	ann03	2.80	3.38	3.94	4.48
	ann04	2.53	3.35	4.22	5.01
	ann05	2.61	3.34	4.10	4.82
	ann06	2.96	3.61	4.28	4.96
	ann07	2.83	3.42	4.09	4.70
	ann08	2.90	3.37	4.15	4.95
	ann09	2.52	3.40	4.11	4.81
	ann10	2.81	3.29	4.07	4.87

RMSE: root mean-square error; *t*_HP_: time horizon of prediction.
